# Spectral and mode properties of surface plasmon polariton waveguides studied by near-field excitation and leakage-mode radiation measurement

**DOI:** 10.1186/1556-276X-9-430

**Published:** 2014-08-25

**Authors:** Ming-Yang Pan, En-Hong Lin, Likarn Wang, Pei-Kuen Wei

**Affiliations:** 1 Research Center for Applied Sciences, Academia Sinica, 128 Academia Road, Section 2, Taipei 115, Taiwan; 2 Institute of Photonics Technologies, National Tsing Hua University, 101 Kuang-Fu Road, Section 2, Hsinchu 300, Taiwan; 3 Department of Optoelectronics, National Taiwan Ocean University, 2 Pei-Ning Road, Keelung 202, Taiwan; 4 Department of Mechanical and Mechatronic Engineering, National Taiwan Ocean University, 2 Pei-Ning Road, Keelung 202, Taiwan

**Keywords:** Surface plasmon polariton, Near-field optics, Nanophotonics, Coupling method, Optical waveguide

## Abstract

We present a method to couple surface plasmon polariton (SPP) guiding mode into dielectric-loaded SPP waveguide (DLSPPW) devices with spectral and mode selectivity. The method combined a transmission-mode near-field spectroscopy to excite the SPP mode and a leakage radiation optical microscope for direct visualization. By using a near-field fiber tip, incident photons with different wavelengths were converted into SPPs at the metal/dielectric interface. Real-time SPP radiation images were taken through leakage radiation images. The wavelength-dependent propagation lengths for silver- and gold-based DLSPPWs were measured and compared. It confirms that silver-based SPP has a propagation length longer than a gold-based one by 1.25, 1.38, and 1.52 times for red, green, and blue photons. The resonant coupling as a function of wavelength in dual DLSPPWs was measured. The coupling lengths measured from leakage radiation images were in good agreement with finite-difference time domain simulations. In addition, the propagation profile due to multi-SPP modes interference was studied by changing position of the fiber tip. In a multimode DLSPPW, SPP was split into two branches with a gap of 2.237 μm when the tip was at the center of the waveguide. It became a zigzag profile when the SPP was excited at the corner of the waveguide.

## Background

Surface plasmon polariton (SPP) waveguides allow electromagnetic wave propagating along metal-dielectric interface with a feature size smaller than optical wavelength. Due to the Ohmic loss of the metal, the propagation length of conventional SPP mode is limited to few microns. There are increasing interests in designing SPP waveguides with a longer propagation length [1-3]. A simple way to increase the SPP length and confine light in subwavelength region is to coat a submicron dielectric strip onto the silver or gold thin film; such dielectric-loaded SPP waveguide (DLSPPW) [4] can increase the length up to tens of microns. Several waveguide devices based on DLSPPWs have been demonstrated, such as waveguide ring resonators [4], interferometers [5], and splitters [6]. The excitation of SPP waveguide modes can be done by both electronic and photonic ways. For example, an electron tunneling current can launch free electrons into SPP mode [7]. By controlling the momentum of free electrons, SPP emission with a spectrum from 650 to 800 nm was demonstrated. For the photonic excitation method, the momentum matching with SPP's propagation constant can be achieved by using attenuated total reflection in an optical prism [8] or grating-coupling effect [9]. A simple way by focusing a laser beam onto the edge of the waveguide can also couple SPPs into waveguides due to the light-scattering effect [10]. The propagation images of SPP modes are often measured by using near-field scanning microscopy [11]. For the above methods, the excitation of SPP modes needs an optical prism and a waveguide coupler to match the SPP momentum. The waveguide device is complicated. The launching position of SPPs is fixed at the end of waveguide, and the focused spot is limited to the diffraction. The launch condition of the SPP mode is hard to be controlled. Besides, the scanning near-field optical measurement is a time-consuming process.

In this paper, we present a near-field excitation system (NFES) to excite the SPP modes. This system provides efficient SPP coupling at any location of the waveguide with various excitation wavelength. The NFES is combined with a leakage radiation microscopy [12] (LRM). It provides direct visualization of the SPP mode in real time. To demonstrate the functions of the proposed setup, we measured different DLSPPW devices. The DLSPPW fabrication is simple. The dielectric stripe can be easily functionalized to provide thermo-optical, electro-optical, or all-optical functionalities for the development of active plasmonic components.

## Methods

**Figure 1:**
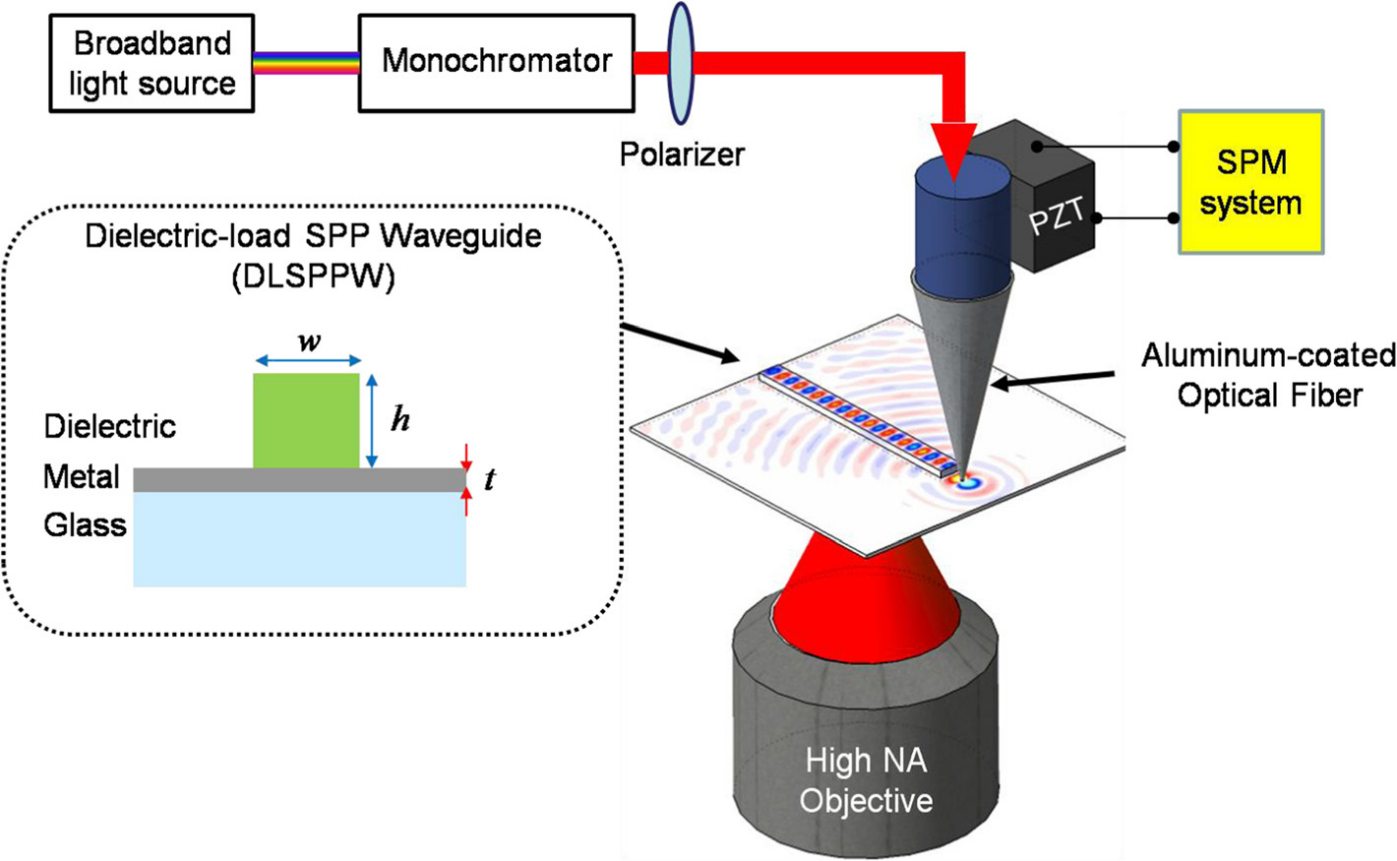
**Schematic setup of a DLSPPW excited by the NFES.** The excitation light was a supercontinuum light and the incident wavelength was selected by a monochromator. The DLSPPW was made of a dielectric strip coated on a metallic thin film on a glass substrate.

The optical setup of NFES is shown in Figure 1. The aluminum-coated tapered fiber tip fabricated by using end-etching process was mounted on an XYZ piezoelectric (PZT) stage. To maintain the optical near-field excitation, the distance between the fiber tip and DLSPPW was controlled by shear-force feedback system and tuning-fork detection method. Broadband light source or monochromatic light selected by a monochromator was coupled into the fiber probe. The subwavelength pinhole at the fiber end converted the guiding wave in the fiber into evanescent wave. Because only transverse magnetic (TM) wave can excite the SPP mode, the incident polarization was also controlled through a linear polarizer to produce evanescent wave with TM polarization. Due to the distance between the tip and SPP waveguide was much smaller than the wavelength, the evanescent wave can be coupled by the waveguide. The large wave vectors of evanescent wave can match momentums of different SPP modes.

The system was used to study the propagation properties of the DLSPPW. The SPP mode in the DLSPPW has a propagation constant *β* = *β*^′^ + *iβ*^″^ with an effective index (*n*_spp_), where *n*_spp_ = *β*/*k*_0_. The effective index is the equivalent refractive index of the surface plasmon waveguide. It depends on the wavelength, modes, dielectric constants of materials, and geometry of the waveguide. That can be calculated by numerical method [13] or determined by Fourier plane analysis [14]. For a dielectric stripe with a refractive index similar to the glass substrate, the *n*_spp_ will be smaller than the index of glass (*n*_g_ = 1.48). The metallic film thickness is smaller than 100 nm; therefore, the SPP mode will have an evanescent tail in the glass substrate. It results in a small leakage of light, radiating at an angle (*θ*) of *sin*^- 1^(*n*_spp_/*n*_g_). The angular wave vector of the leakage radiation is the same as *n*_spp_ and larger than air. Conventional optical microscope with an air lens cannot image the SPP mode. In the system, we applied a high numerical aperture (NA = 1.45) oil objective. The 1.45 NA is larger than the *n*_spp_ which can collect the leakage radiation from the SPP mode. The intensity distribution of the leakage light is proportional to the SPP mode profile. Therefore, the propagation properties of SPP mode in the DLSPPW can be directly observed by recoding the leakage radiation images from a CCD camera.

Additional file 1 shows an example of a DLSPPW excited by using NFES and observed by the LRM. The excitation wavelength was 633 nm. The DLSPPW had a waveguide width (*w*) of 400 nm and waveguide height (*h*) of 500 nm, and the thickness of the silver (*t*) was 100 nm. The narrow dielectric strip of the DLSPPW was made of an electron beam photoresist (ma-N2403, MicroResist Technology, Berlin, Germany). It is transparent in the visible to near-infrared region and has a refractive index about 1.61. The bright spot in the video shows the optical field at the fiber tip. The tip location was manipulated by the PZT stage. In the experiment, the fiber tip was first located at the corner of waveguide. It excited a zigzag pattern due to the reflection from both sides of the waveguide. The fiber tip was moved from the corner to the middle of the waveguide. The zigzag pattern became a dashed straight line. The pattern was resulted from the interference of the lowest two modes in the waveguide [15].

Additional file 2 shows the NFES operated in wavelength scanning mode. The fiber tip was fixed at the end of a DLSPPW. This waveguide width (*w*) was 300 nm, waveguide height (*h*) 300 nm, and thickness of the silver (*t*) 100 nm. It supported single SPP mode at a longer wavelength and became a multimode waveguide at a shorter wavelength. The color CCD recorded red straight light pattern for single SPP mode. It showed blue beating pattern due to multimode interference of SPP modes. The movies verify the advantages of the NFES and LRM methods for real-time plasmonic waveguide characterization with tunable wavelength and excitation positions. With this system, the propagation properties of DLPPWs with different metallic films, dielectric coatings, and layouts were studied and compared.

## Results and discussion

### Propagation length of DLSPPW

**Figure 2:**
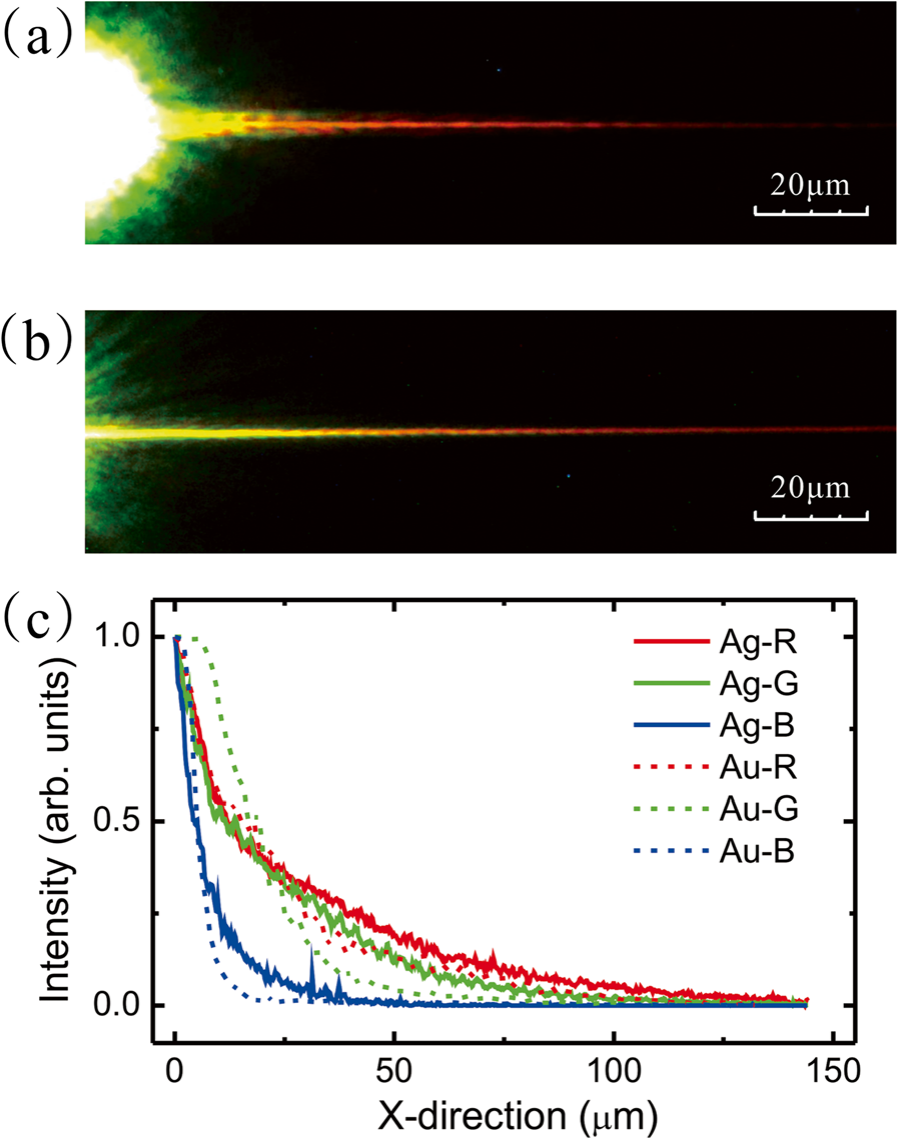
**Leakage radiation images and intensity profiles of DLSPPW for gold-based and silver-based DLSPPWs.** Leakage radiation images of SPPs on **(a)** gold film and **(b)** silver film. The bright spot is the excitation source from the fiber tip. **(c)** The SPP intensities as a function of distance as measured at RGB pixels in a color CCD.

The properties of guiding broadband SPPs in DLSPPW with different metal films were studied by the setup. The dielectric strip was 200-nm wide and 300-nm high which coated on 100-nm-thick gold and silver films. DLSPPWs were excited directly by a white light source without the monochromator. Figure 2a,b shows the color CCD images of the leakage radiation of SPP mode on gold and silver films, respectively. In both cases, the propagation lengths of SPPs with red color were much longer than green and blue ones. The intensity of leakage radiation was proportional to the intensity in the waveguide. Therefore, we can measure the propagation loss directly from the images. The electric field of SPPs is written as *E*(*z*) = *E*_0_*e*^*iβx*^. The propagation length *L* defined by the distance of SPPs intensity decay to a factor of 1/*e* can be written as *L* = 1/2*β*^″^, where the decay constant $\beta^{''}=\frac{2\pi }{\lambda }\text{Im}\left(n_{\text{spp}}\right)$. The propagation length is dependent on the imaginary part of dielectric constant of materials and geometry of the waveguide. We obtain the *L* by fitting the measurement intensity by the equation *I* = *I*_0_ + *Ae*^-*x*/*L*^. Figure 2c shows the RGB intensities as a function of propagation distance. Compared with the propagation length in gold-based DLSPPW and silver-based one, the propagation length of the silver film was 1.25, 1.38, and 1.52 times longer than gold-based SPP at red, green, and blue color, respectively. The dielectric constants are -7.0124 + 0.2119*i*, -11.626 + 0.3634*i*, and -18.096 + 0.4842*i* for silver and -1.7562 + 5.2986*i*, -4.5461 + 2.4577*i*, and -11.548 + 1.2821*i* for gold at wavelengths of 450, 530, and 630 nm, respectively. These wavelengths are corresponding to the peak wavelengths of RGB pixels in the CCD. It can be found that the imaginary parts of dielectric constants of silver are much smaller than those of gold. It indicates that silver has a longer propagation length than gold at the same wavelength. In addition, the propagation length of gold-based SPPs is increased from blue to red light because the imaginary part of dielectric constant is substantially decreased. Therefore, the ratio between the propagation lengths in silver- and gold-based waveguides is increased from red to blue light. The measured phenomenon is consistent with the wavelength-dependent dielectric constants of silver and gold.

### Multimode interference

**Figure 3:**
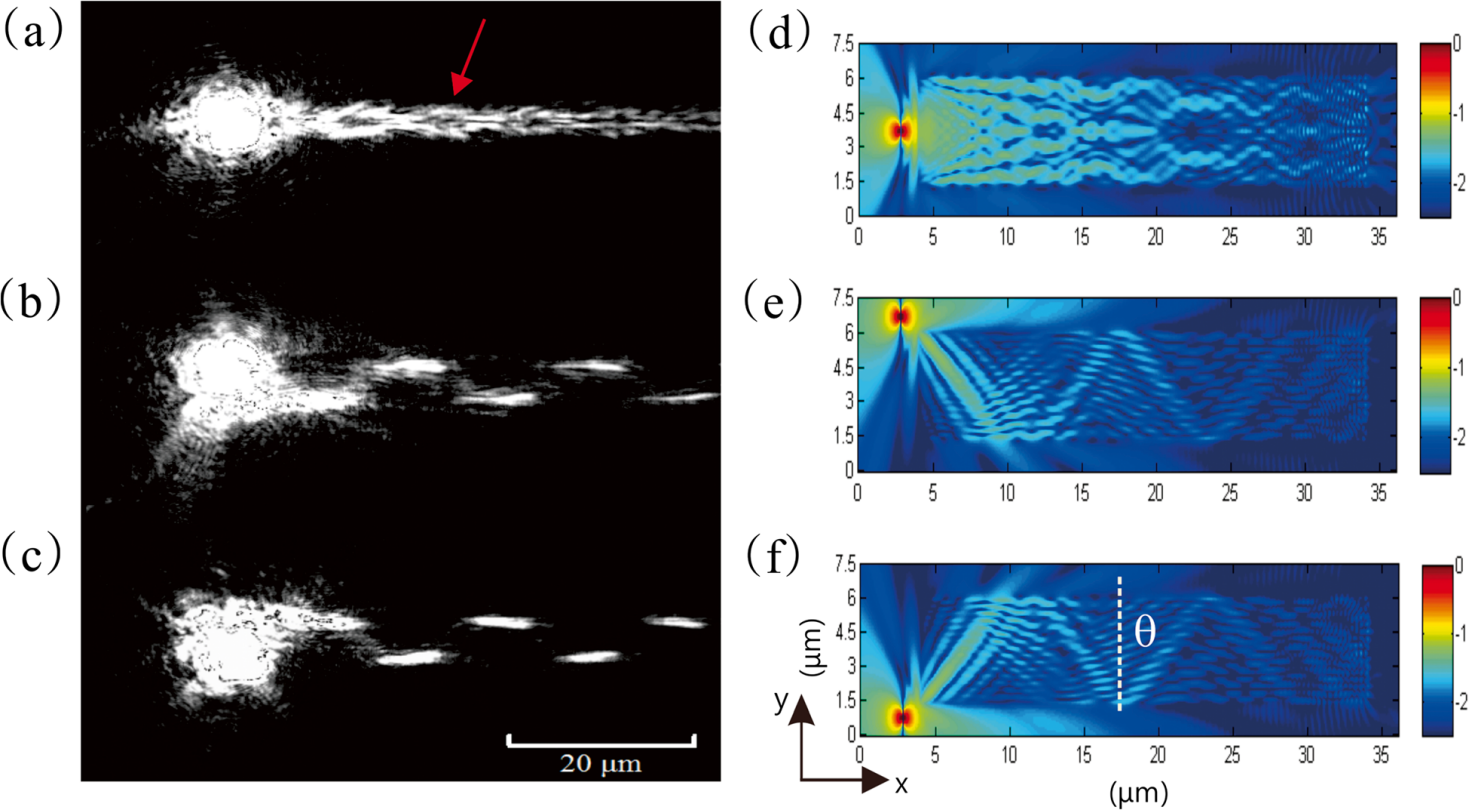
**A multimode waveguide excited by NFES. (a)** Leakage radiation image when the fiber tip was at the center of the waveguide. The red arrow shows the location of intensity was spitted into 50:50. **(b, c)** Leakage radiation images when the fiber tip was located at two different corners of the waveguide. **(d to f)** The calculated optical field distributions (*E*_*z*_) for near-field excitation at different positions, **(d)** at the center of waveguide, **(e, f)** and at two different corners.

Multimode interference (MMI) is a waveguide effect which waveguide modes are interfered and self-imaged in a multimode waveguide. MMI is used for the plasmonic couplers due to its large fabrication tolerance and integrated size [16,17]. Several works have presented plasmonic MMI couplers to split SPP intensities and filter wavelengths. Multimode interference couplers were often studied using calculation methods, such as finite-element method [18] (FEM), beam propagation method [17] (BPM), and finite-difference time-domain (FDTD) method [19]. By using these methods, the functions of MMI devices can be theoretically demonstrated. However, MMI patterns are hard to be directly visualized. To show MMI in DLSPPW experimentally, we studied a wide DLSPPW with 300-nm-high, 4.6-μm-wide strip on a 100-nm-thick silver film. The waveguide length was longer than 100 μm. The incident wavelength was 830 nm owing to the good SPP propagation length and quantum efficiency of CCD. This waveguide provided TM_00_ ~ TM_06_ in 830-nm wavelength and gave rise to multimode interference along the waveguide. The interference effect can be express by

(1) $$E_{z}\left(x,\;y\right)=\overset{6}{\underset{m=0}{\sum }}a_{m}u_{m}\left(y\right)e^{-j\beta_{m}x}$$

where *m* is the number of guided mode, *a*_*m*_ is superposition constant and *u*_*m*_(*y*) is complex amplitude depended with incident field. The MMI pattern changed with the incident field *u*_*m*_(*y*).The incident field was changed by varying the launching position of the fiber tip. In the experiment, the near-field excitation location was moved from the north corner to south corner by using move-mode in NFES. Figures 3 show the leakage radiation images that correspond the fiber tips located at corners and middle of the waveguide. Figure 3a was a chain-like MMI pattern. The field intensity was splitting to 50:50 with a gap of 2.237 μm (red arrow). Figure 3b,c shows the LRM images when input field was launched at the corner. Both of them showed zigzag bright dashed lines and symmetric to each other. Some inconspicuous illuminations were observed between these bright patterns. The angle of refraction is about 40°.

**Figure 4:**
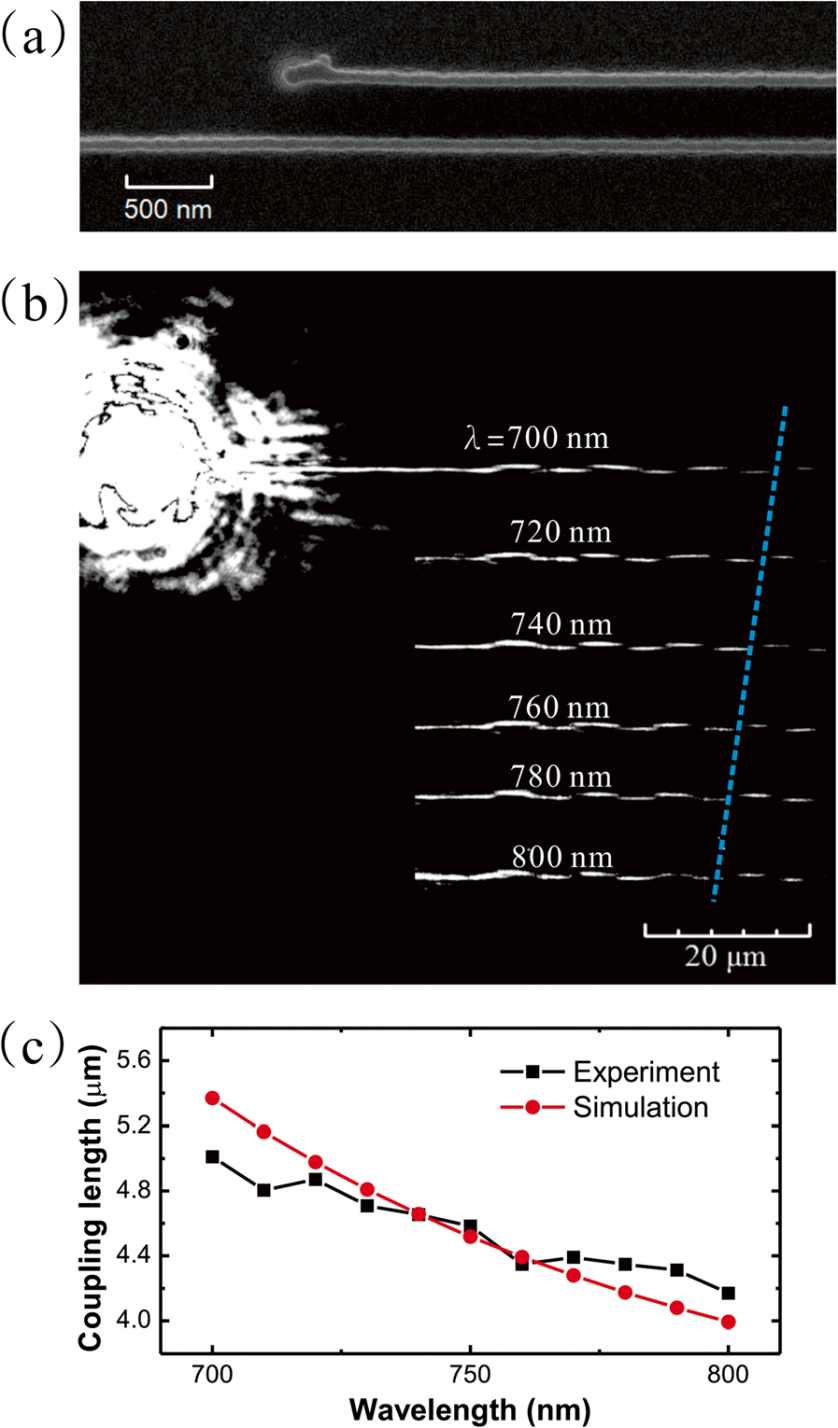
**Dual DLSPPW coupler studied by NFES with different wavelengths. (a)** SEM image of DLSPPW-based dual waveguides coupler. **(b)** Leakage radiation images of SPP waves propagation in the coupler from *λ* = 700 to 800 nm wavelengths. Cyan dash line showed the coupling length was decreased with the incident wavelength. **(c)** The measured and calculated coupling lengths as a function of wavelength. Red line shows the calculation results. Black line shows the measured results.

To understand these properties, we calculated the plasmonic modes (*E*_*z*_) by using 3D-FDTD method. The calculation fields were shown in Figure 3d,e,f). In these simulations, a 300-nm-hight, 4.6-μm-width, and 30-μm-length dielectric stripe with a refractive index of 1.61 was placed on 100-nm-thick silver film coated on a glass substrate. A near-field source was set near the dielectric stripe at different lateral positions of the waveguide. Figure 3d shows a MMI pattern generated by middle-launch configuration. Near-field source launch evanescent field coupled into the waveguide and then formed interference patterns. Input intensity was split into 50:50 at a position of *x* = 21 μm with gap 2.1 μm, which is very close to the experimental result (2.237 μm). Moreover, the simulated propagation length is 15.87 μm, which is qualitative agreement with the experimental result, 13.82 μm. It is noted that this waveguide is too short to support self-imaging effect.Simulations of corner-launched configurations were shown in Figure 3e,f. That was corresponding to experimentally result of Figure 4b,c, respectively. First, concentrated field was distributed at the corner near the light source, then the field split into three paths and guided following at specific angles. These angles correspond to wavevector components. Ray-optic-like effect was observed by analyzing the main path. The reflection angle of the simulation is about 43.5°. A difference is found in corner-launch cases when compared with experimental result. The intensity of leakage radiation at the edge of the waveguide is brighter than inside the region, but it is invisible in simulation. This effect is attributed to the scattering effect by the rough waveguide sidewalls. The intensity of leakage radiation is weaker than scattering light so the bright patterns were observed at the waveguide sidewalls.

### Dual DLSPPW coupler

When two waveguides are very close to each other, their mode fields overlap and optical energy is transferred from one waveguide to the other. This dual waveguide coupler has been applied for many kinds of devices, such as power splitter, wavelength filter, and optical modulator. Understanding the coupling property is an important issue in the applications. The proposed setup can be well applied to the measurement of the plasmonic coupling between dual DLSPPWs. Figure 4a shows a scanning electron microscopy (SEM) image of a dual DLSPPW coupler. The coupler was consisted of two 90-nm wide and 300-nm high DLSPPW, which supported only fundamental TM_00_ mode at wavelengths from *λ* = 480 to 800 nm. The gap of both waveguides was 420 nm. Figure 4b shows the leakage radiation images of SPP mode from *λ* = 700 to 800 nm wavelengths. Due to the directional coupling effect, period oscillation of the SPP mode was observed. The coupling length (*L*_c_) was defined by the length needed for optical power transferred from one waveguide to the other. As indicated in the blue dash line in Figure 4b, the coupling length decreased with the increase of excited wavelength. The coupling length in a dual DLSPPW coupler can be considered as a symmetric and an anti-symmetric modes propagating in the coupler with different propagation constants *β*_+_ and *β*_-_[20]. The phase shift *φ*_±_ is *β*_±_*L*, where *L* is the propagation distance. Mode power in one of waveguide will transfer to the other waveguide when Δ*φ* = *φ*_+_ - *φ*_-_ = *π*. The coupling length is defined as the distance for the *π* phase difference, $L_{c}=\frac{\pi }{\Delta \beta }=\frac{\lambda }{2\Delta n_{\text{eff}}}$ where Δ*β* = *β*_+_ - *β*_-_, *Δn*_spp_ = *n*_spp+_ - *n*_spp-_. Since the *L*_c_ is related to *n*_spp_. It will depend on the wavelength, modes, dielectric constants of materials, and geometry of the waveguide. The reason is that increase of the wavelength will increase the SPP mode size. It has a longer evanescent tail overlapping between neighboring waveguides. The coupling becomes stronger; thus, the coupling length is shorter. To verify the measurement of propagation properties in the directional coupler, both symmetric and asymmetric modes, the mode solver through vector finite-difference method was used. We found the coupling length, *L*_c_ = 5.37 μm at wavelength *λ* = 700 nm. The length was decreased to *L*_c_ = 3.99 μm at wavelength *λ* = 800 nm. Figure 4c shows the comparison between the measured and calculated results. The results are in good agreement between calculated lengths and the measured leakage radiation images.

## Conclusions

We proposed a new optical setup that provides tunable spectral and modal excitation for surface plasmon polariton waveguide. The SPP images with broadband and single wavelength excitation at different excitation positions were demonstrated. The waveguides with different layouts and materials can be quickly compared by this setup. We confirmed the better SPP mode for longer wavelength excitation on silver film-based waveguides. The coupling length of dual plasmonic coupler was studied by using tunable wavelength mode. An increase of SPP coupling with the increase of wavelength was observed and identified with the calculation results. This setup takes advantages of nanoscale excitation, lower background, wavelength selectivity, and controllable excitation positions for direct visualization. In addition to the proposed DLSPPW devices, this technique can be applied to study other types of plasmonic waveguides and devices, such as ring oscillators [21], interferometers [22], plasmonic logic gates [23], etc.

## Competing interests

The authors declare that they have no competing interests.

## Authors' contributions

MYP, EHL, and PKW designed the near-field excitation system. MYP fabricated and measured all the samples in this article. MYP, LW, and PKW performed data analysis. All the authors read and approved the final manuscript.

## Supplementary Material

Additional file 1 **Leakage radiation images of SPP waves.** Leakage radiation images of SPP waves when the fiber tip was moved along the width direction of the waveguide. Click here for file

Additional file 2 **Leakage radiation images of SPP waves.** Leakage radiation images of SPP waves when the incident wavelength was scanned from red to blue wavelength. Click here for file
